# Visualization of endogenous gut bacteria in *Drosophila melanogaster* using fluorescence *in situ* hybridization

**DOI:** 10.1371/journal.pone.0247376

**Published:** 2021-02-19

**Authors:** Irfan Akhtar, Fiona A. Stewart, Anna Härle, Andrea Droste, Mathias Beller

**Affiliations:** 1 Institut für Mathematische Modellierung Biologischer Systeme, Heinrich-Heine-Universität Düsseldorf, Düsseldorf, Germany; 2 Systembiologie des Fettstoffwechsels, Heinrich-Heine-Universität Düsseldorf, Düsseldorf, Germany; University of Salento, ITALY

## Abstract

All metazoans are colonized by a complex and diverse set of microorganisms. The microbes colonize all parts of the body and are especially abundant in the gastrointestinal tract, where they constitute the gut microbiome. The fruit fly *Drosophila melanogaster* turned out to be an exquisite model organism to functionally test the importance of an intact gut microbiome. Still, however, fundamental questions remain unanswered. For example, it is unknown whether a fine-tuned regionalization of the gut microbiome exists and how such a spatial organization could be established. In order to pave the way for answering this question, we generated an optimized and adapted fluorescence *in situ* hybridization (FISH) protocol. We focused on the detection of the two major *Drosophila* gut microbiome constituting bacteria genera: *Acetobacter* and *Lactobacillus*. FISH allows to detect the bacteria *in situ* and thus to investigate their spatial localization in respect to the host as well as to other microbiome members. We demonstrate the applicability of the protocol using a diverse set of sample types.

## Introduction

Microbiome research has become an emerging and central field in biology and biomedicine. This is based on the fact that all metazoans are colonized by billions of microorganisms, which deeply impact the overall physiology of the host. The human body, for example, consists of 3 x 10^13^ cells, but harbors another 4 x 10^13^ microorganisms of enormous diversity [1]. The majority of bacteria colonize the gut, where they not only facilitate nutrient access and uptake, but also e.g. protect from pathogens or are important to maintain the intestinal barrier function of the gut [2]. Through a variety of enzymes and metabolic pathways the gut microorganisms are able to metabolize complex molecules and thus make them available for the host [3]. Further, certain metabolites which cannot be synthesized by the host itself can be provided by its gut microbiome [4]. A dysbiosis of the gut microbiome accordingly results in serious health problems and is also associated with the occurrence and progression of severe chronic diseases such as obesity, inflammatory bowel disease, diabetes mellitus, nonalcoholic fatty liver disease (NAFLD) and hepatocellular carcinoma [5–7].

Many gut microbiome members cannot be cultured in the lab. Yet, investigations targeting the functional importance of the gut microbiome require the ability to query the presence of distinct bacterial species. Thus, molecular biology techniques including (quantitative) PCR and next generation sequencing are routinely used to probe the microbiome composition under various conditions. A shortcoming of these methods is that they lack spatial information both in relation to the host as well as to other microorganisms. The fluorescence *in situ* hybridization (FISH) method overcomes this shortcoming and was previously used to detect e.g. the bacteria in human saliva [8]. The FISH method relies on fluorescently labeled probes, which hybridize to their specific DNA or RNA sequence targets [9,10]. For microbiome applications, those target sequences are usually regions of the 16S rRNA of the given gut bacterium.

*Drosophila melanogaster* is commonly used to study various aspects of the gut microbiome. So far, however, FISH was not extensively applied to probe for the spatial organization of the bacteria present in the gut. This is intriguing, as the low complexity of the fly microbiome with only 10 to 20 bacterial species should be well suited. While one study applied FISH to investigate the symbiont/pathogen *Wolbachia* and the gut microbiome member *Acetobacter pasteurianus* in gnotobiotic animals [11] and one study used the eubacteria probe Eub338 with flies [12], most microbiome studies in *Drosophila melanogaster* either utilized genetically modified, fluorescent versions of gut bacteria [13], or used tracers to test for e.g. the vitality of the gut bacteria [14]. Those methods have the draw-back that they either require the addition of exogenous, genetically modified bacteria, which could alter their abundance and location in the gut, or the lack of species differentiation. We thus generated an adapted and optimized protocol to add FISH as a method to probe the microbiome composition of *Drosophila*.

## Materials and methods

### Drosophila fly husbandry

All experiments were performed with *w1118* (*white[–]*) flies, which were maintained at 25°C with 60–70% humidity and a 12 h light/dark cycle. Flies were kept on food containing 8% yeast extract, 8% cornmeal, 0.8% agar, 0.4% propionic acid, 0.15% nipagin.

### Fluorescence *in situ* hybridization probes

The used probes for FISH (Table 1) consisted of a generic probe, which should detect all eubacteria (Eub338; S-D-Bact-0338-a-A-18) [15,16], a probe which specifically detects bacteria of the genus *Lactobacillus* (Lacto722; S-G-Lacb-0722-a-A-25) [16,17], and a third probe which hybridizes with the genus *Acetobacter* (Aceto). The sequences for the Eub338 and the Lacto722 probes were obtained from *Probebase* (http://probebase.csb.univie.ac.at/node/8). For the design of the Aceto probe we used the webtool *decipher* (http://www2.decipher.codes/DesignProbes.html).

**Table 1:** FISH probes used in the present study.

Probe	Sequence 5’→3’	Specificity	Fluorophore	Excitation (Ex) / Emission (Em) for LSM710	Excitation (Ex) / Emission (Em) for Operetta CLS
Eub338	GCTGCCTCCCGTAGGAGT	Eubacteria	Atto425	Ex: 405 nm	Ex: 355–385 nm
				Em: 464–489 nm	Em: 430–500 nm
Aceto	CGCCTTTGACCCTCAGG	*Acetobacter*	Atto488	Ex: 488 nm	Ex: 460–490 nm
				Em: 501–551 nm	Em: 500–550 nm
Lacto722	YCACCGCTACACATGRAGTTCCACT	*Lactobacillus*	Atto594	Ex: 633 nm	Ex: 530–560 nm
				Em: 641–755 nm	Em: 570–650 nm

As the Eub338 probe generated high levels of background signal in the feces samples (S1A Fig) as well as in the *Drosophila* larval and adult guts, we only used this probe as a control with the type strain bacteria to check whether the strain-specific Aceto and Lacto722 probes result in distinct hybridization with the corresponding bacteria. All probes were ordered from Biomers (https://www.biomers.net/) as “DOPE” double labeled fluorophore probes (each of the double labeled oligos carries one fluorescent label at the 5’- and 3’-end, respectively).

### Fluorescence *in situ* hybridization of bacterial cell suspensions

The basis for the herein established method was a FISH protocol described by Valm *et al*., (2011). To establish the protocol and to test for specificity of the probes, bacterial cells of type strains (Table 2) grown as overnight cultures were used.

**Table 2:** Bacterial strains used in the present study.

DSMZ No.	Strain	Gram Type	Cultivation condition
6897	*Escherichia coli* K12 DH5α	negative	37°C, LB medium, aerobic
3509	*Acetobacter pasteurianus*	negative	26–28°C, YPM medium, aerobic
15551	*Acetobacter tropicalis*	negative	30°C, Acetic Acid bacterium medium (AABM), aerobic
20174	*Lactobacillus plantarum*	positive	30°C, MRS medium, microaerophilic
20054	*Lactobacillus brevis*	positive	30°C, MRS medium, microaerophilic
20203	*Lactobacillus fructivorans*	positive	30°C, MRS medium, microaerophilic

**Fig 1:**
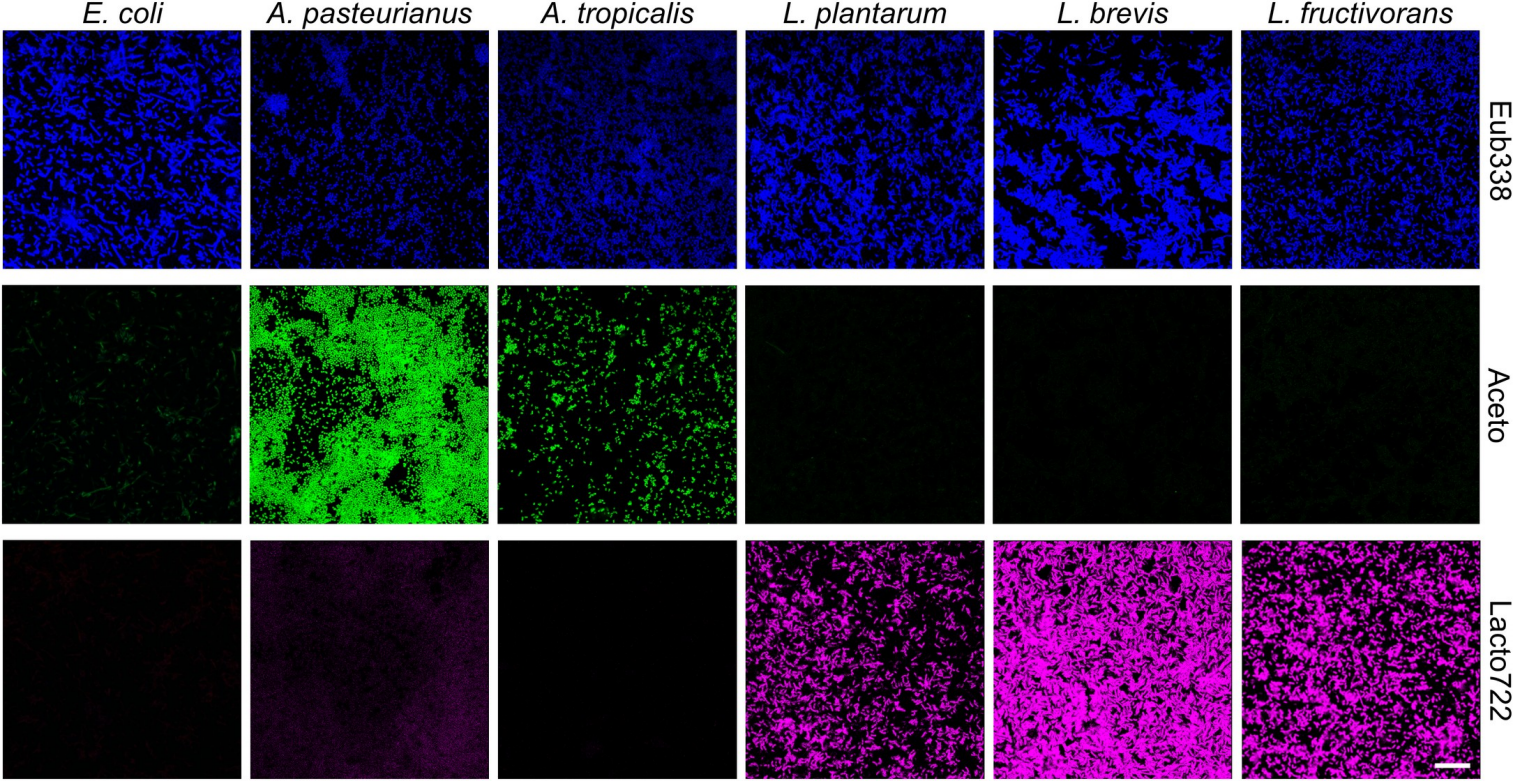
Validation of the probe specificity for fluorescence *in situ* hybridization (FISH) using bacterial cell suspensions. FISH was performed with PFA-fixed bacterial cell suspensions of *E*. *coli*, *A*. *pasteurianus*, *A*. *tropicalis*, *L*. *plantarum*, *L*. *brevis*, and *L*. *fructivorans* with the three different probes Eub338 (blue), Aceto (green), and Lacto722 (red) (4 μM/probe) and 40% formamide for three hours at 46°C. *Lactobacillus* strains were treated with 10 mg/ml lysozyme for 15 minutes at 37°C prior to the hybridization. Each bacterial species sample was individually stained with the respective probe and the figure only shows the corresponding images for either the Eub338, Aceto or Lacto722 hybridization channel. All images were recorded with the identical settings for each probe channel. To assay for possible fluorescence bleed-through, we recorded for all stainings all three channels (S6 Fig). The figure shows representative examples from at least three separate experiments. Scalebar represents 20 μm.

The staining experiments were performed in standard reaction tubes. After determining the OD_600_ of the given bacterial culture via photometry, the cells were fixed in 500 μL 5% paraformaldehyde solution (pH 7) (10% 10x PBS, 10% EGTA [0.5 M] pH 8, 10% paraformaldehyde, diluted 1:2 with 1x PBS) for 15 minutes. After fixation, the cells were washed twice with PBS (8% NaCl, 0.2% KCl, 1.44% Na_2_HPO_4_, 0.24% KH_2_PO_4_ pH 7.4). If needed, the cells can be stored in a mixture of PBS and 100% ethanol (1:1 mixture) at -20°C. The cells were then treated with 10 mg/mL lysozyme in PBS for 15 minutes at 37°C. The fixed cells were suspended in 100 μL hybridization buffer (5.255% NaCl, 10% Tris-HCl [0.2 M] pH 7.5, 0.1% SDS, 40% formamide, 4 μM FISH probe each), and incubated for three hours at 46°C. After hybridization, the cells were washed with wash buffer 2 (5.255% NaCl, 10% Tris-HCl [0.2 M] pH 7.5, 0.1% SDS) for 30 minutes at 48°C. For DNA staining we either used the TO-PRO-1 or TO-PRO-3 stains (Molecular Probes / Invitrogen / Thermo Scientific) diluted 1:1000 in the wash buffer 2 washing step. Afterwards, the cells were resuspended in 400 μl resuspension buffer (0.146% NaCl, 10% Tris-HCl [0.2 M] pH 7.5). For plate reader analyses (e.g. S1B Fig), the resuspended bacteria were distributed as quadruplicates in 96-well plates (OptiPlate-96F, Perkin Elmer). FISH staining intensity was normalized to the TO-PRO staining. For microscopic analyses (e.g. Fig 1), 10 μL bacterial suspension was spotted on microscope slides. After drying at room temperature, the sample was covered with 30 μL Prolong Gold Antifade reagent (Thermo Fisher Scientific) and a coverslip. Samples were imaged with a Zeiss LSM710 microscope with the settings provided in Table 1 and a 63x oil objective.

### Fluorescence *in situ* hybridization of isolated *Drosophila* gut bacteria

For the isolation of the *Drosophila* gut bacteria about 30 six-day old male flies were kept at -20°C for 10 minutes. Afterwards the flies were put in a reaction tube and washed with 1 ml of a 10% bleach solution followed by two additional washing steps with 1 ml 70% EtOH and 1 ml sterile PBS to remove external bacteria. Afterwards, the whole flies were homogenized in 200 μl sterile PBS using a tissue grinder (Kontes^TM^ Pellet Pestle^TM^ Motor, Kimble Chase). After homogenization, the tube was filled up with PBS to 1 ml. This solution was diluted 1:10 and plated on either MRS plates (1% casein peptone, 1% meat extract, 0.5% yeast extract, 2.2% glucose X H_2_O, 0.1% Tween80 [1.06 g/ml], 0.2625% K_2_HPO_4_ X 3H_2_O, 0.5% sodium acetate, 0.2% ammonium citrate, 0.02% MgSO_4_ X 7H_2_O, 0.005% MnSO_4_ X H_2_O, 1.5% agar, pH 6.2–6.5), YPM plates (2.5% mannitol, 0.3% peptone, 0.5% yeast extract, 1.2% agar), or ACE plates (1% glucose, 1.5% casein peptone, 0.8% yeast extract, 1.5% agar) and incubated for three days at 28°C. For each plate type (MRS, ACE and YPM), we washed off the colonies with the corresponding liquid medium (thus, e.g. MRS liquid medium was used to wash off the colonies from the MRS agar plates and corresponding procedures were performed for the ACE and YPM samples). These resuspended bacteria colonies were then incubated over night at 28°C. Subsequently, the cells were processed as described in the section “Fluorescence in situ hybridization of bacterial cell suspensions”. Samples were imaged with an Operetta CLS high content screening microscope (Perkin Elmer) with the settings provided in Table 1 and a 40x air objective.

### Fluorescence *in situ* hybridization of *Drosophila* feces

To detect and visualize bacteria in the feces of adult *Drosophila*, about 30 flies of mixed age were transferred to new food vials which were then covered with a microscope glass slide. The flies were kept in the vial and on the glass slide for about 24 hours, after which the glass slide was removed. The feces, which were visible on the slide, were dried at 50°C on a thermal block for 2 hours. All further steps were performed directly on the glass slide. The feces were fixed with 500 μL 5% paraformaldehyde solution for 15 minutes and were afterwards washed with PBS and treated with 10 mg/mL lysozyme in PBS for 15 minutes at 37°C. Then the hybridization buffer (including 40% formamide and 4 μM probe each) was layered over the feces and incubated for three hours at 46°C. The feces were afterwards washed in wash buffer 2 for 30 minutes at 48°C and finally washed with resuspension buffer. The buffer was removed, and the feces left to dry completely before they were covered with 30 μl Prolong Gold Antifade reagent and a cover slip. Samples were imaged with an Operetta CLS high content screening microscope (Perkin Elmer) with the settings provided in Table 1 and a 40x air objective.

### Generation of axenic *Drosophila* animals

The protocol to generate axenic animals was adapted from [18]. In brief, adult *Drosophila* animals were placed in a fly cage, which was covered with an apple juice agar plate (for approximately 20 plates: 20 g agar, 8.5 g sucrose, 500 ml dH_2_O, 170 ml naturally cloudy apple juice, 10 ml Nipagin solution [700 ml 96% EtOH p.a., 300 ml dH_2_O, 100 g Nipagin]) with a dab of fresh yeast. After 24 h, the apple juice agar plate was replaced by a fresh one. After another 24 h, *Drosophila* embryos were washed off of the agar plate using embryowash (1 ml Triton X-100, 14 g NaCl, 200 ml dH_2_O, sterile filtration) and collected in a 50 ml centrifugation tube. Afterwards, the embryos were dechorionated using 50% bleach for 2 minutes, followed by a washing step with dH_2_O. The tube was centrifuged for 15 seconds at 300 rpm to pellet the embryos. Finally, the embryos were washed with 70% EtOH p.a., followed by another washing step with dH_2_O. Embryos were resuspended in 200 μl embryowash and 20 μl were pipetted onto axenic food vials [19].

### Genomic DNA (gDNA) extraction and PCR-based validation of axenic state

Genomic DNA (gDNA) was isolated from single adult Drosophila flies which were either conventionally reared or axenic. Single flies were placed in an Eppendorf tube and homogenized in 50 μl squishing buffer (10 mM Tris-HCl [pH 8.2], 1 mM EDTA, 25 mM NaCl, 200 μg/ml proteinase K). The homogenate was incubated for 30 minutes at 37°C prior to heat inactivation of the proteinase K at 95°C for 2 minutes. The homogenate was centrifuged for 1 minute at 13.000 rpm and the supernatant was transferred to a new reaction tube. For the PCR, 1 μl of the supernatant and primers targeting the 16S rRNA gene (S-D-Bact-0341-b-S-17:5’ CCTACGGGNGGCWGCAG 3’ and S-D-Bact-0785-a-A-21: 5’ ACTACHVGGGTATCTAATCC 3’ [20]) were used. A typical PCR result is shown in S2 Fig.

### Fluorescence *in situ* hybridization of *Drosophila* larval and adult guts

*Drosophila white[–]* flies were kept on a diet containing 8% yeast extract (to avoid yeast-based autofluorescence signal), 8% cornmeal, 0.8% agar, 0.4% propionic acid and 0.15% nipagin. For the larval samples, wandering third instar larvae were isolated and dissected. For the adult samples, virgin male flies were collected and aged for six days prior to the experiment. The flies were then starved overnight on 0.5% agarose and then refed with bacteria-containing yeast extract/cornmeal diet to motivate food uptake for about 4 hours prior to dissection. Axenic *Drosophila* animals were kept on the same yeast extract / cornmeal-based food, which only contained in addition antibiotics (ampicillin, erythromycin, kanamycin, and tetracyclin [50 μg/ml as final concentration/antibiotic]) and processed identically to the conventionally reared flies.

Guts of wandering third instar larvae and six days old adult flies were dissected in ice-cold PBS and fixed in Carnoy’s solution (60% ethanol, 30% chloroform, 10% acetic acid) for 5 minutes. The guts were washed twice in PBS and treated with 10 mg/mL lysozyme in PBS for 15 minutes at 37°C. As controls, we included experiments where either any probe was omitted in the subsequent hybridization (S3 Fig) or where the guts were pre-treated with 50μg / mL RNase A for 30 minutes prior to the hybridization with the probes (S4 Fig). The tissues were subsequently incubated in hybridization buffer containing 40% formamide with 4 μM of each probe. Hybridization was allowed to happen for about 16 hours at 46°C. The guts were subsequently washed in 500 μl wash buffer 2 for 30 minutes at 48°C. Then the wash buffer 2 was removed and 500 μl resuspension buffer were added. The guts were mounted on microscope slides in 30 μl Prolong Gold Antifade reagent. Samples were imaged with an Operetta CLS high content screening microscope (Perkin Elmer) with the settings provided in Table 1. The overview images of the guts were recorded as tile scans with a 5x air objective. The high-resolution zoom-in images were recorded with a 40x air objective.

### Quantitative analysis of microscopy data

During method optimization, we followed the impact of varying parameters (e.g. probe and formamide concentration) on the staining intensity of the type strain stainings by recording microscopic images and quantifying the staining results via image segmentation. An overview of the image segmentation routine carried out with the KNIME data analysis platform and the image segmentation module is found in S5 Fig. We used the TO-PRO-3 DNA stain as general bacterial marker and then calculated the relative staining efficiencies of the given probes in response to the different parameter variations. The analysis pipeline with example images is deposited in the KNIME pipeline hub and can be accessed at the following URL: https://hub.knime.com/matbeller/spaces/Beller-Laboratory/latest/Akhtar_et_al_Bacterial_FISH_Example_Workflow.

## Results

The goal of the present study was to establish a FISH protocol for *Drosophila melanogaster*. Thus, we started selecting and designing probes for the most abundant bacterial genera living in *Drosophila* guts: *Acetobacter* and *Lactobacillus*. As a control, we used the universal bacterial probe Eub338 (S-D-Bact-0338-a-A-18) (see Table 1), which binds to the 16S rRNA sequence of all eubacteria [15,16]. The *Lactobacillus* specific Lacto722 (S-G-Lacb-0722-a-A-25) probe was obtained from the probe-database *Probebase* (http://probebase.csb.univie.ac.at/node/8) and also targets the 16S rRNA sequence as does the *Acetobacter*-specific probe which we designed using the webtool *decipher* (http://www2.decipher.codes/DesignProbes.html).

### Validation of the FISH probe specificity using bacterial type strains and isolated *Drosophila* gut bacteria

We started with the protocol optimization by testing several critical parameters of the FISH protocol such as the probe concentration (we used 2 μM, 4 μM, 8 μM and 16 μM), the formamide concentration (10%, 20%, 30% and 40%) as well as different fixation times (15, 30, 60 and 90 minutes). The various tests were quantified with a plate reader analysis (S1B Fig) and an automated image analysis pipeline (S5 Fig) and resulted in the selection of a probe concentration of 4 μM, a formamide concentration of 40% and 15 minutes of fixation (for details see material and methods). Longer fixation times resulted in a prominent decrease in signal (S1B Fig). In order to increase the signal intensity for the gram-positive *Lactobacillus*, we also tested whether a treatment with 10 mg/ml lysozyme for 15 minutes at 37°C could enhance the probe signal, which indeed was the case. Therefore, we decided to include this step in every experiment.

The specificity of the strain-specific probes was first tested on bacterial cultures of *E*. *coli* and of various *Lactobacillus* and *Acetobacter* type strains (see Table 2). All probes were tested with all type strains individually to test for potential cross-reactivities. *Lactobacillus* and *Acetobacter* representatives are typical members of the *Drosophila* gut microbiome [21,22]. Fig 1 displays exemplary results of FISH stainings with bacterial cells from liquid cultures which were hybridized with each of the three probes.

**Fig 2:**
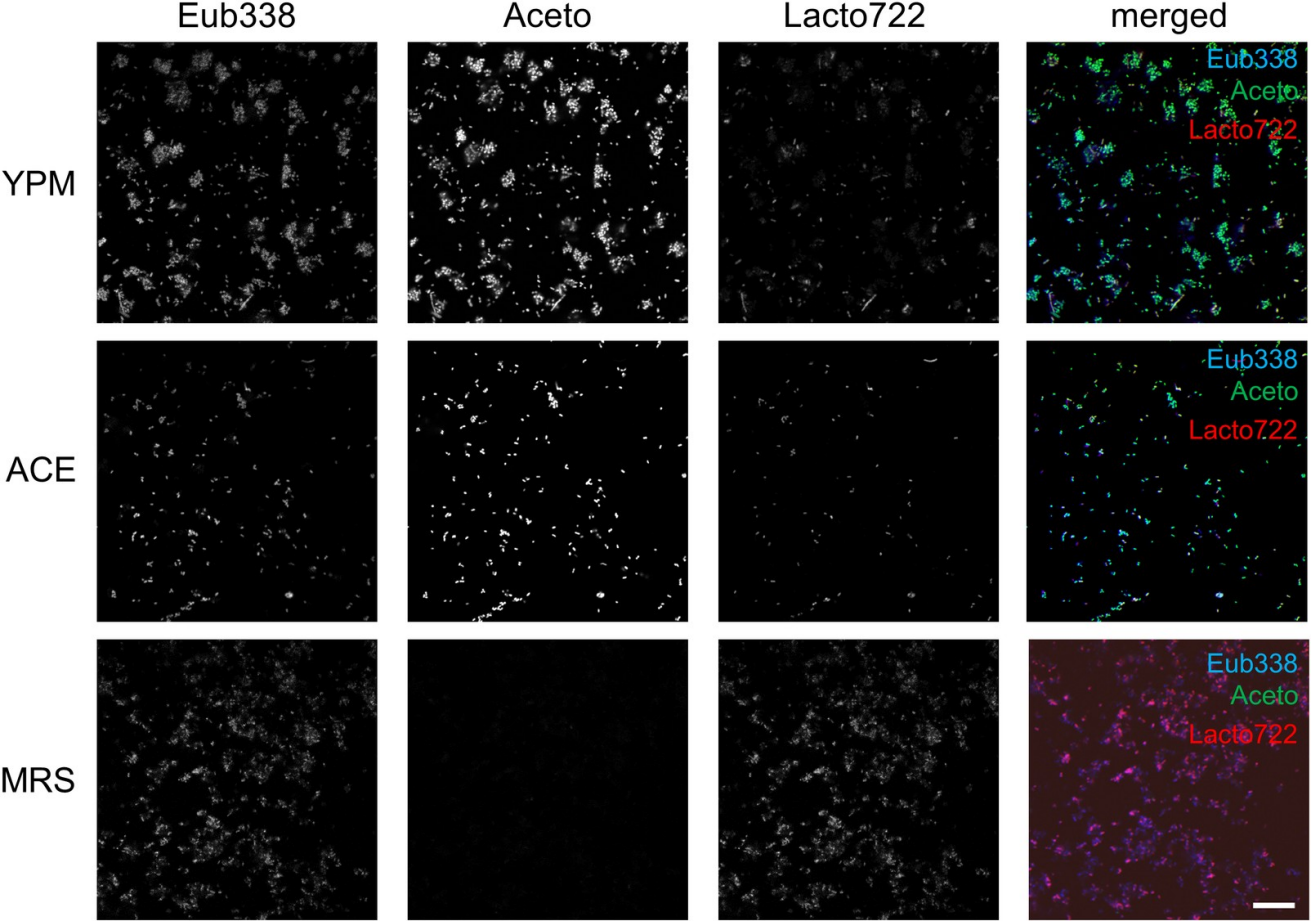
Fluorescence *in situ* hybridization with isolated *Drosophila* gut microbiomes. FISH was performed with PFA-fixed isolated *Drosophila* gut bacteria using the three different probes Eub338 (blue), Aceto (green), and Lacto722 (red) (4 μM/probe) and 40% formamide for three hours at 46°C. The isolates were treated with 10 mg/ml lysozyme for 15 minutes at 37°C prior to the hybridization. Bacteria were cultured in either YPM-, ACE- or MRS-medium. The figure shows representative examples from at least three separate experiments. Scalebar represents 20 μm.

The universal Eub338 probe hybridized with all tested bacterial species, whereas the *Lactobacillus* and *Acetobacter* probes only showed a strong hybridization signal with the specific species (Fig 1). Both genus specific probes only showed minute hybridization signals with *E*. *coli* cells. In order to test for a potential bleed-through fluorescence across the channels as well as for a putative background fluorescence of a given bacterial species, we recorded in each single probe staining experiment all three channels with the identical settings (S6 Fig). No bleed-through signals or prominent background fluorescence signals were present. Thus, our initial tests demonstrate the specificity of the utilized probes and specificity of the detection by the absence of fluorescence bleed-through. In order to further test the applicability of the probes, and to make sure that the probes work with the bacteria present in the *Drosophila* gut, we tested them on unpurified bacterial cultures (Fig 2) that we previously isolated from adult *Drosophila* flies using *Lactobacillus* (MRS) and *Acetobacter* (YPM, ACE) enriching agar plates (see material and methods). Here, the bacteria were isolated from whole flies (see material and methods for details) and the mix of bacteria which grew on the respective enrichment media agar plate was used for the staining experiment. This way, we could make sure that the probes do not only detect the type strain representatives (Fig 1), but also the bacteria present in *Drosophila* (Fig 2).

Using the unpurified strains obtained from YPM and ACE plates, the signal of the Aceto-probe was much stronger as compared to the Lacto722 probe (Fig 2). FISH staining with the MRS isolates resulted in the juxtaposed results, as expected. The eubacteria Eub338 probe resulted in a prominent signal in all isolates and all bacteria could be detected. Thus, our probes in combination with the protocol used by us are capable to detect *Drosophila* resident bacteria.

### FISH with *Drosophila* feces

**Fig 3:**
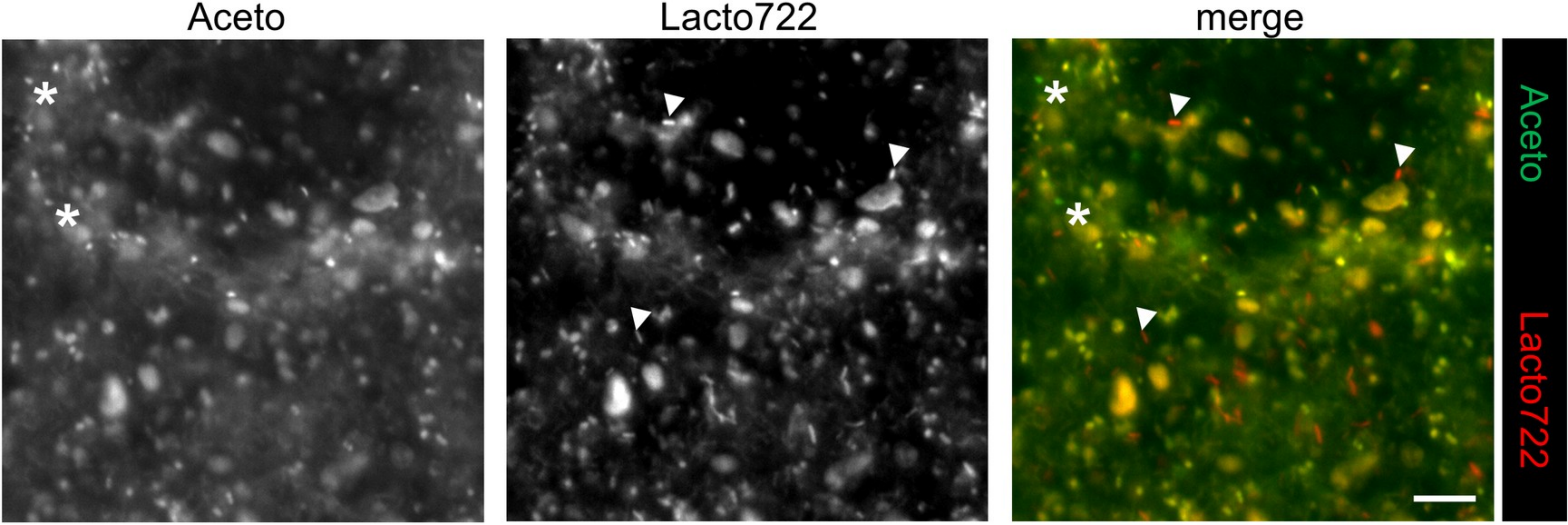
Fluorescence *in situ* hybridization of adult *Drosophila* feces. FISH was performed with PFA-fixed adult *Drosophila* feces using the genera-specific probes Aceto (green) and Lacto722 (red) (4 μM/probe) and 40% formamide for three hours at 46°C. Prior to hybridization, feces samples were treated with 10 mg/ml lysozyme for 15 minutes at 37°C. Detected *Acetobacter* cells are marked with asterisks and *Lactobacillus* cells are indicated by arrowheads. The figure shows representative examples from at least three separate experiments. Scalebar represents 5 μm.

Next, we tested whether it is possible to visualize gut bacteria in feces of adult *Drosophila* flies. The generic Eub338 probe hybridized with many bacteria present in the feces samples. However, the Eub338 probe also resulted in high background signals, presumably based on an unspecific binding to food remnants (S1A Fig). Thus, we decided to only move on with the strain-specific Aceto and Lacto722 probes. Fig 3 shows a typical FISH staining of *Drosophila* feces collected from adult flies (see material and methods). Both the Aceto and the Lacto722 probe hybridized with bacteria dispersed in between remnants of the food, which resulted in some background signal. Based on the morphology and staining properties, however, specific signals for both bacterial genera could be identified (see asterisks and arrowheads in Fig 3).

### FISH with larval and adult *Drosophila* guts

The final aim of establishing the staining protocol was to perform FISH on bacteria localized in the *Drosophila* gut. In pilot experiments, we realized that a standard paraformaldehyde (PFA)-based fixation, which we used for the isolated bacteria (Fig 2) and the feces (Fig 3), resulted in low fluorescence intensities (S7i Fig). One alternative fixative is the organic solvent-based Carnoy’s solution ([23] and material and methods). PFA-fixed *Drosophila* guts stained with the DNA stain TO-PRO-3 only showed a signal in the gut epithelium (S7i Fig). The same staining following a fixation with Carnoy’s solution (see material and methods) resulted in much higher staining intensities, also allowing the detection of luminal bacteria (S7ii and S7iii Fig). Further, also the morphology of the gut was better retained, as for example also the mucosa layer of the gut now became visible (S7iv Fig).

**Fig 4:**
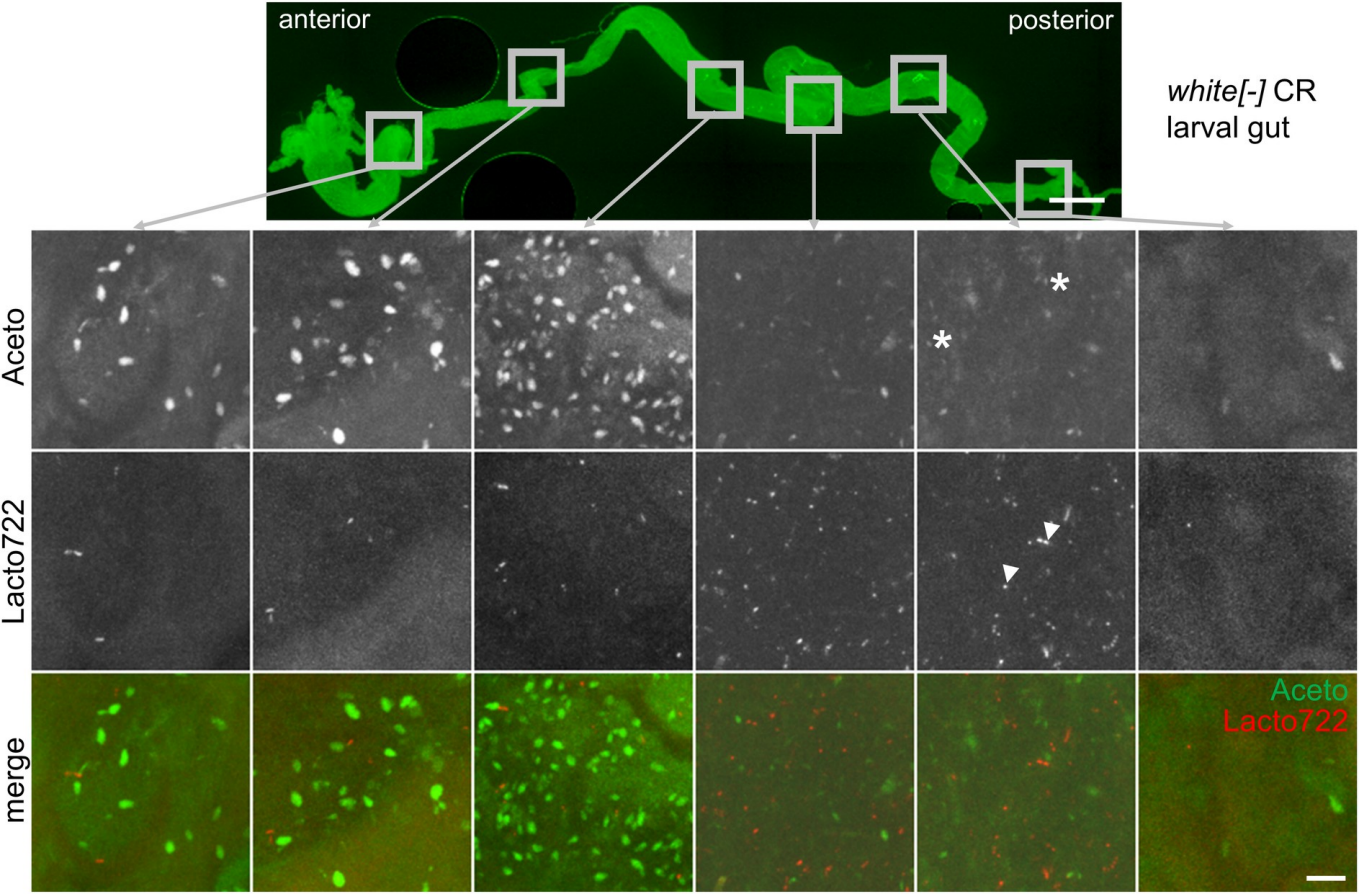
Fluorescence *in situ* hybridization of larval *Drosophila* guts. FISH was performed with Carnoy’s solution-fixed larval *Drosophila* guts using the genera-specific probes Aceto (green) and Lacto722 (red) (4 μM/probe) and 40% formamide for 16 hours at 46°C. Prior to hybridization, larval guts were treated with 10 mg/ml lysozyme for 15 minutes at 37°C. An overview of the entire gut was imaged and detailed zoom-ins of 6 different regions are shown. The figure shows representative examples from at least three separate experiments. Exemplary *Acetobacter* cells are marked with asterisks and *Lactobacillus* cells are indicated by arrowheads. In each experiment 5 to 10 guts per condition were dissected. Scalebars represent 500 μm (overview) and 10 μm (zoom-ins).

We first started with FISH stainings on larval guts of wandering third instar animals. The staining procedure resulted in the detection of both *Lactobacillus* and *Acetobacter* cells (Fig 4). Especially in the green spectrum, however, a prominent background signal was present, likely originating from food present in the gut lumen as well as tissue autofluorescence. As for the feces samples, a differentiation based on the morphology and size between the bacteria and background signals was nevertheless possible. In order to test further for the specificity of the staining, we also performed control stainings without probes (S3 Fig) or with RNase A pre-treatment (S4 Fig) with guts of conventionally reared larvae as well as FISH with guts of axenic larvae (S8 Fig). Across all control stainings a weak and mostly homogeneous background fluorescence was visible which again was most prominent in the green channel. When the control stainings (S3, S4 and S8 Figs) are compared to the FISH stainings performed with the conventionally reared larvae (Fig 4), however, only the latter showed prominent and distinct signals, thus demonstrating specificity of the staining.

As *Drosophila* larvae are constant feeders, the detection of food-borne bacteria is relatively easy. However, adult *Drosophila* are intermittent feeders [24] which potentially complicates the timing for the detection of bacteria in the gut. Therefore, we starved the six-day old animals overnight and afterwards put them on food vials containing bacteria for four hours, prior to dissection and analysis of the adult guts.

**Fig 5:**
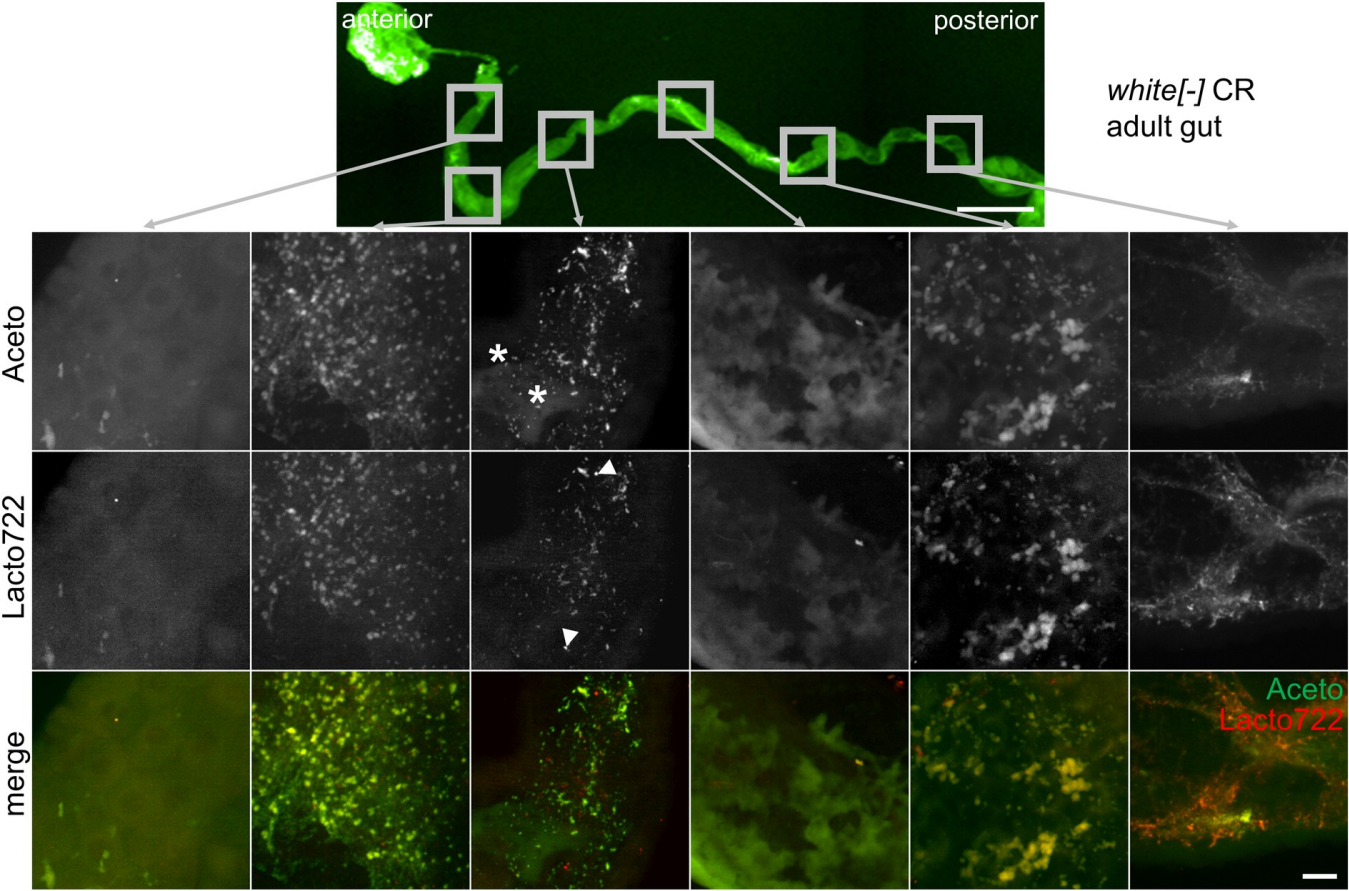
Fluorescence *in situ* hybridization of adult *Drosophila* guts. FISH was performed with Carnoy’s solution-fixed adult *Drosophila* guts using the genera-specific probes Aceto (green) and Lacto722 (red) (4 μM/probe) and 40% formamide for 16 hours at 46°C. Prior to hybridization, larval guts were treated with 10 mg/ml lysozyme for 15 minutes at 37°C. An overview of the entire gut was imaged and detailed zoom-ins of six different regions are shown. The figure shows representative examples from at least three separate experiments. Exemplary *Acetobacter* cells are marked with asterisks and *Lactobacillus* cells are indicated by arrowheads. In each experiment 5 to 10 guts per condition were dissected. Scalebars represent 500 μm (overview) and 10 μm (zoom-ins).

This regimen allowed the detection of both *Acetobacter* and *Lactobacillus* in adult fly guts (Fig 5). Axenic animals did not show any clear-cut signals (S9 Fig). Our initial staining results demonstrate specific signals for both *Acetobacter* and *Lactobacillus*. Whether a clear-cut regionalization of the gut colonization is present, future experiments need to show.

## Discussion

In this study, we describe a method to detect *Acetobacter* and *Lactobacillus*, two of the most abundant commensal *Drosophila* gut bacteria [21,22], in *Drosophila* larval and adult fly guts as well as in *Drosophila* feces and for isolated gut bacteria using fluorescence *in situ* hybridization (FISH). Many studies aiming at the analysis of gut microbiota apply (quantitative) polymerase chain reaction ((q)PCR) [25,26] or next generation sequencing (NGS) [27] to query the microbiome composition and abundance of certain species. Both methods, however, fail to provide spatial information. FISH in contrast enables the spatially precise and genus-specific distinction of bacteria. This has multiple advantages such as the analysis of a potential preference of the one or the other bacterial species for a certain gut compartment or a co-occurrence of certain bacteria in the form of consortia. As an exchange of metabolites between gut microbiome members of *Drosophila* was demonstrated [28,29] such a co-occurrence seems likely. When, where and how the bacteria interact or whether the metabolites instead are exchanged via a long distance, however, is not yet fully understood. A potential caveat of the FISH staining procedure and the dissection of the gut is that both manipulations potentially interfere with the normal bacteria distribution. Yet, the procedure is probably still closer to the physiological situation as compared to feeding genetically-modified fluorescent versions of the gut bacteria in large amounts to axenic animals in order to generate gnotobiotic variants.

Bacterial FISH has been used to investigate the microbiome in other insects such as beetles [30], the Asian citrus psyllid [31] or *Drosophila suzukii* [32] and is a standard method for corresponding studies in mammals [33–35]. Yet, in *Drosophila* only one study so far utilized the Eub338 probe to detect endogenous bacteria in the gut [12] and one study detected *Acetobacter* and *Wolbachia* in gnotobiotic animals [11]. Here, we present protocols suited for the detection of *Drosophila* gut bacteria in culture, in feces or in the gut of larvae or adult animals. During the design of our experiments, we realized that FISH protocol parameters vary widely between studies. To determine suitable staining parameters, we tuned multiple critical parameters such as the formamide concentration in the hybridization buffer, the probe concentration as well as the fixative and fixation time. For the sake of simplicity, we determined the effect of parameter variations in stainings of pure bacteria cultures and by quantifying the staining results using plate-reader as well as microscopy and image-segmentation based methods (S1 and S5 Figs). A parameter untouched by us was the number of fluorophores per probe and variations of the fluorophores themselves. Changing these parameters might allow an even more sensitive detection of bacteria as well as beneficial staining characteristics, as especially in the green channel background fluorescence of the food and the tissue in the larval and adult samples could be noted (S3, S4, S8 and S9 Figs). The biggest impact in our experiments had the fixation time and the fixative used. Previous FISH protocols performed with tissue samples mostly utilized a PFA-fixation, sometimes combined with an alcoholic dehydration prior to the actual hybridization (e.g. [12]). In our experience, the fixation with Carnoy’s solution resulted in a superior signal to background behavior and better preservation of the overall morphology of the gut than the PFA-fixation (S7ii-S7iv Fig). Several previous reports state that the use of PFA for fixing guts results in the entire loss or collapse of the mucus layer [34,36,37] which is in line with our observations. Carnoy’s was also used before for various staining protocols including FISH in invertebrates [31], yet often with much longer fixation times. The fixative performance likely depends on multiple parameters such as the fixation and hybridization time and the presence or absence of e.g. an additional dehydration step.

Altogether, our protocol presented herein should serve as a starting point for future experiments and provide a guideline for optimizing e.g. novel probes capable to differentiate bacteria down to the species level. Expanding the range of detected bacteria by multiplexing with such additional probes and assaying for the impact of environmental or genetic perturbations on the gut microbiome organization will pave the way to a better understanding of the gut microbiome compartmentalization and interactivity.

## Supporting information

S1 Fig Impact of fixation time on the labeling intensity and the application of the Eub338 probe on *Drosophila* feces samples. (A) FISH with *Drosophila* feces and the Eub338 (blue), Aceto (green) and Lacto772 (red) probes (4 μM/probe) and 40% formamide for three hours at 46°C. Prior to hybridization, feces samples were treated with 10 mg/ml lysozyme for 15 minutes at 37°C. The images exhibit a representative example from at least three independent experiments. The scalebar in (A) represents 5 μm. The Eub338 probe showed a strong background staining. (B) *E*. *coli* cells were paraformaldehyde fixed for the given timespans prior to hybridization with 4 μM of the Eub338 probe. Fluorescence signal was detected with a Synergy Mx plate reader (BioTek) and normalized to the signal of the TO-PRO-3 DNA stain. Bars show mean values of quadruplicate measurements and error bars represent standard deviation.
(TIF)

S2 Fig PCR confirmation of the axenic state. The agarose gel picture shows an exemplary 16S rRNA PCR (see material and methods) with DNA from a six-day old conventionally reared (CR) male *white[–]* fly (with microbiome) and the DNA of three individual six-day old male axenic *white[–]* flies (lacking a microbiome). The water control is additionally shown. Only in the CR sample the expected amplicon of about 500 bp length is present.
(TIF)

S3 Fig No probe control fluorescence *in situ* hybridization of larval *Drosophila* guts. FISH was performed with conventionally reared (CR) larval *Drosophila* guts isolated from *white[–]* animals. While the standard hybridization conditions of 40% formamide for 16 hours at 46°C was used, no probes were added. The upper part of the figure shows an overview of the entire gut. Zoom-in views of six different regions are shown below. 5 to 10 guts per condition were dissected. Scalebars represent 500 μm (overview) and 10 μm (zoom-ins).
(TIF)

S4 Fig Fluorescence *in situ* hybridization of RNase A-treated larval *Drosophila* guts. FISH was performed with conventionally reared (CR) larval *Drosophila* guts isolated from *white[–]* animals using the genera-specific probes Aceto (green) and Lacto722 (red) (4 μM/probe) and 40% formamide for 16 hours at 46°C. Prior to hybridization, larval guts were treated with 10 mg/ml lysozyme for 15 minutes and 50 μg/mL RNase A for 30 minutes at 37°C. The upper part of the figure shows an overview of the entire gut. Zoom-in views of six different regions are shown below. 5 to 10 guts per condition were dissected. Scalebars represent 500 μm (overview) and 10 μm (zoom-ins).
(TIF)

S5 Fig FISH image analysis pipeline. Flow-chart of the image segmentation pipeline for the quantitative analysis of the impact of various parameters on the FISH staining efficiency of singular bacterial type strain stainings. On the left an overview of the varied parameters during method optimization is given. On the right-hand side an overview of the image segmentation procedure performed with the KNIME image analysis platform is provided. The complete analysis pipeline and example images are provided at the KNIME hub (https://hub.knime.com/; see material and methods). (A) and (B) refer to separate analysis routines for the Eub338 and TO-PRO signals.
(TIF)

S6 Fig Probe and fluorescence detection specificity tests using various bacteria. FISH was performed with PFA-fixed bacterial cell suspensions of *E*. *coli*, *A*. *pasteurianus*, *A*. *tropicalis*, *L*. *plantarum*, *L*. *brevis*, and *L*. *fructivorans* with the three different probes Eub338 (blue–detection in the DAPI channel), Aceto (green–detection in the GFP channel), and Lacto722 (red–detection in the RFP channel) (4 μM/probe) and 40% formamide for three hours at 46°C. *Lactobacillus* strains were treated with 10 mg/ml lysozyme for 15 minutes at 37°C prior to the hybridization. While each bacterial species sample was only stained with one probe (A: Eub388, B: Aceto, C: Lacto722) all detection channels were imaged with constant settings in order to test for potential fluorescence bleed-through. The figure shows representative examples from at least three separate experiments. Scalebar represents 20 μm.
(TIF)

S7 Fig Fixation of larval *Drosophila* guts using PFA and Carnoy’s solution. Larval *Drosophila* guts were fixed for 15 minutes using PFA (i) and for 5 minutes with Carnoy’s solution (ii-iv). DNA was stained with TO-PRO-3. (i) and (ii) were imaged using the same microscope settings and show the much higher staining intensity using the Carnoy’s solution. (iii) TO-PRO-3 stained bacteria in larval *Drosophila* gut lumen. (iv) Zoom-in of (iii) showing gut bacteria marked by arrowheads. The asterisk highlights the mucosa layer. Scalebars represent 100 μm (i-ii), 50 μm (iii), and 20 μm (iv).
(TIF)

S8 Fig Fluorescence *in situ* hybridization of axenic larval *Drosophila* guts. FISH was performed with axenic larval *Drosophila* guts using the genera-specific probes Aceto (green) and Lacto722 (red) (4 μM/probe) and 40% formamide for 16 hours at 46°C. Prior to hybridization, larval guts were treated with 10 mg/ml lysozyme for 15 minutes at 37°C. An overview of the entire gut was imaged and detailed zoom-ins of six different regions are shown. The figure shows representative examples from at least three separate experiments. In each experiment 5 to 10 guts per condition were dissected. Scalebars represent 500 μm (overview) and 10 μm (zoom-ins).
(TIF)

S9 Fig Fluorescence *in situ* hybridization of axenic adult *Drosophila* guts. FISH was performed with axenic adult *Drosophila* guts using the genera-specific probes Aceto (green) and Lacto722 (red) (4 μM/probe) and 40% formamide for 16 hours at 46°C. Prior to hybridization, guts were treated with 10 mg/ml lysozyme for 15 minutes at 37°C. An overview of the entire gut was imaged and detailed zoom-ins of six different regions are shown. The figure shows representative examples from at least three separate experiments. In each experiment 5 to 10 guts per condition were dissected. Scalebars represent 500 μm (overview) and 10 μm (zoom-ins).
(TIF)
