# Supplementary figures and images for: Visualization of endogenous gut bacteria in Drosophila melanogaster using fluorescence in situ hybridization
Source: PLoS One. 2021 Feb 19;16(2):e0247376. doi: 10.1371/journal.pone.0247376 (PMC7894962; doi:10.1371/journal.pone.0247376)

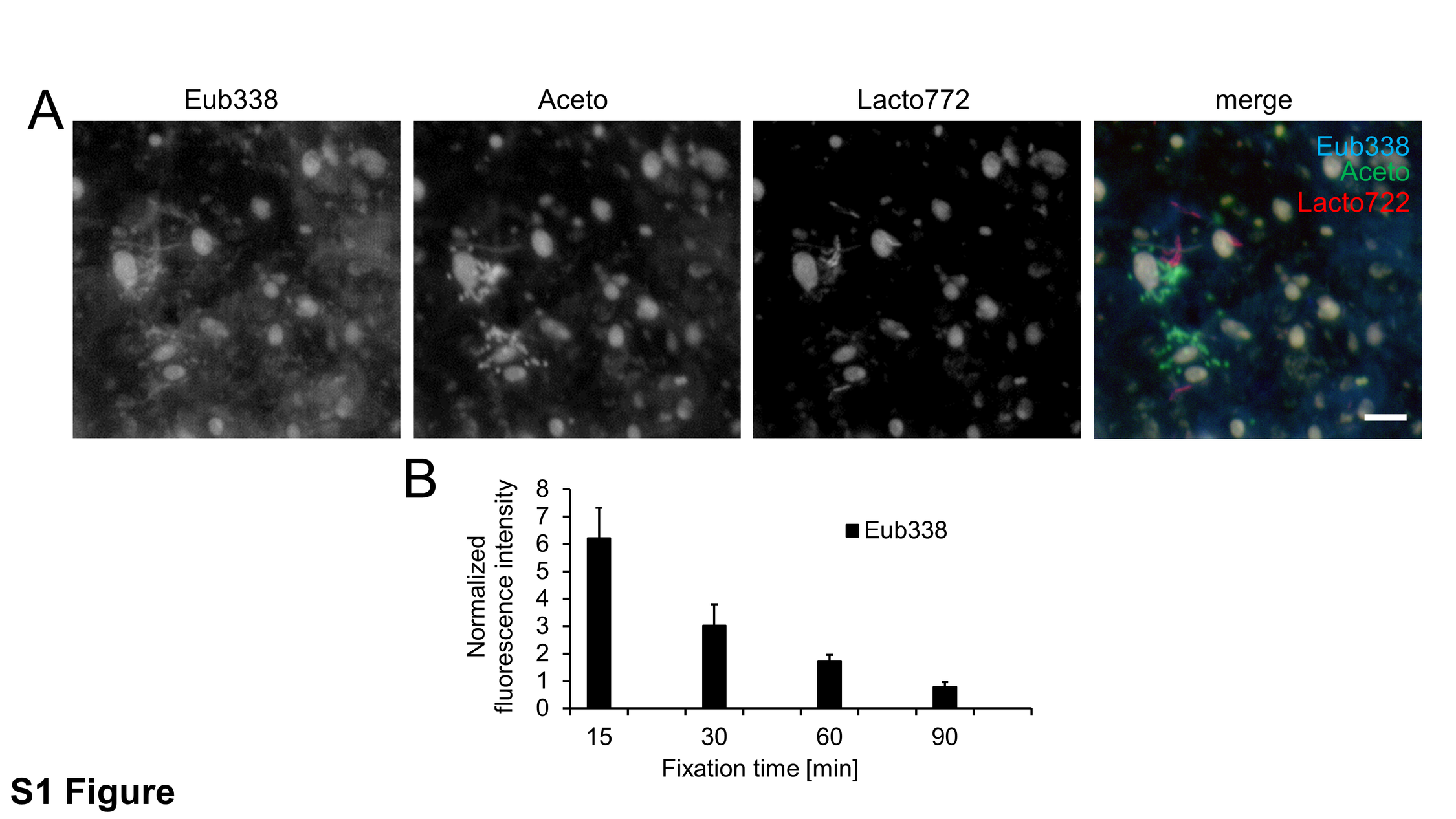

Supplement: S1 Fig — (A) FISH with Drosophila feces and the Eub338 (blue), Aceto (green) and Lacto772 (red) probes (4 μM/probe) and 40% formamide for three hours at 46°C. Prior to hybridization, feces samples were treated with 10 mg/ml lysozyme for 15 minutes at 37°C. The images exhibit a representative example from at least three independent experiments. The scalebar in (A) represents 5 μm. The Eub338 probe showed a strong background staining. (B) E. coli cells were paraformaldehyde fixed for the given timespans prior to hybridization with 4 μM of the Eub338 probe. Fluorescence signal was detected with a Synergy Mx plate reader (BioTek) and normalized to the signal of the TO-PRO-3 DNA stain. Bars show mean values of quadruplicate measurements and error bars represent standard deviation. (TIF) [file pone.0247376.s001.tif]

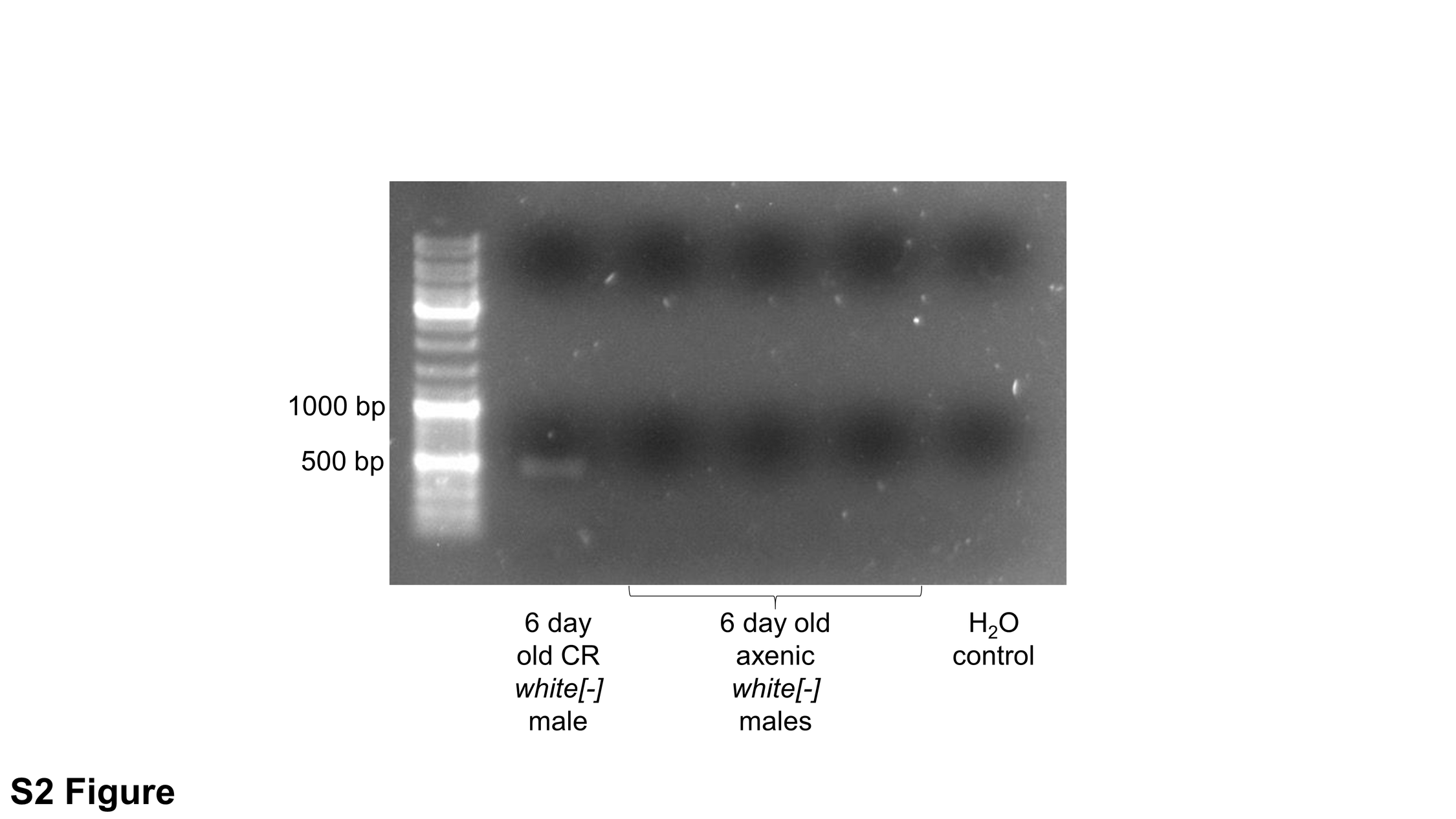

Supplement: S2 Fig — The agarose gel picture shows an exemplary 16S rRNA PCR (see material and methods) with DNA from a six-day old conventionally reared (CR) male white[–] fly (with microbiome) and the DNA of three individual six-day old male axenic white[–] flies (lacking a microbiome). The water control is additionally shown. Only in the CR sample the expected amplicon of about 500 bp length is present. (TIF) [file pone.0247376.s002.tif]

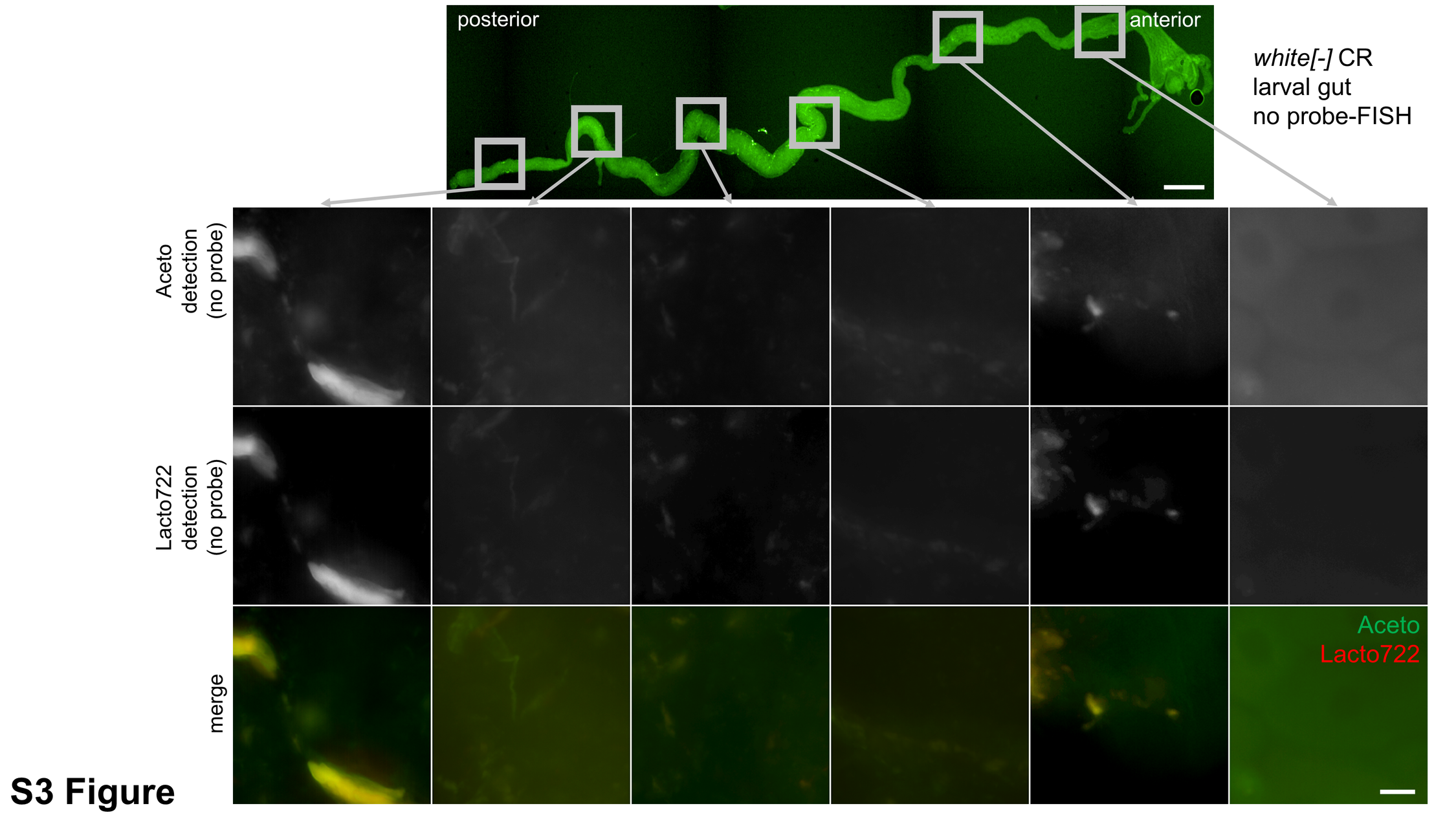

Supplement: S3 Fig — FISH was performed with conventionally reared (CR) larval Drosophila guts isolated from white[–] animals. While the standard hybridization conditions of 40% formamide for 16 hours at 46°C was used, no probes were added. The upper part of the figure shows an overview of the entire gut. Zoom-in views of six different regions are shown below. 5 to 10 guts per condition were dissected. Scalebars represent 500 μm (overview) and 10 μm (zoom-ins). (TIF) [file pone.0247376.s003.tif]

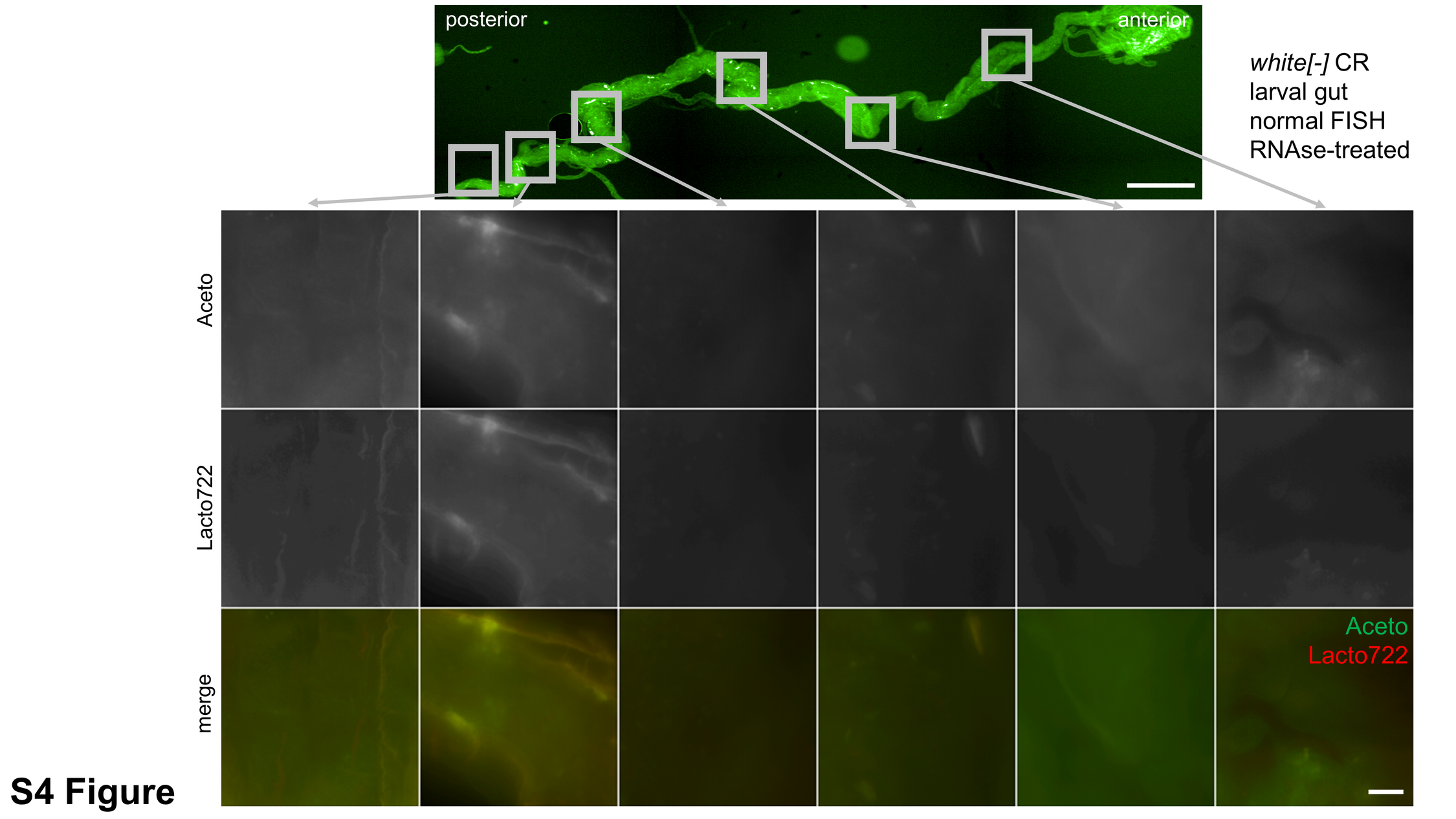

Supplement: S4 Fig — FISH was performed with conventionally reared (CR) larval Drosophila guts isolated from white[–] animals using the genera-specific probes Aceto (green) and Lacto722 (red) (4 μM/probe) and 40% formamide for 16 hours at 46°C. Prior to hybridization, larval guts were treated with 10 mg/ml lysozyme for 15 minutes and 50 μg/mL RNase A for 30 minutes at 37°C. The upper part of the figure shows an overview of the entire gut. Zoom-in views of six different regions are shown below. 5 to 10 guts per condition were dissected. Scalebars represent 500 μm (overview) and 10 μm (zoom-ins). (TIF) [file pone.0247376.s004.tif]

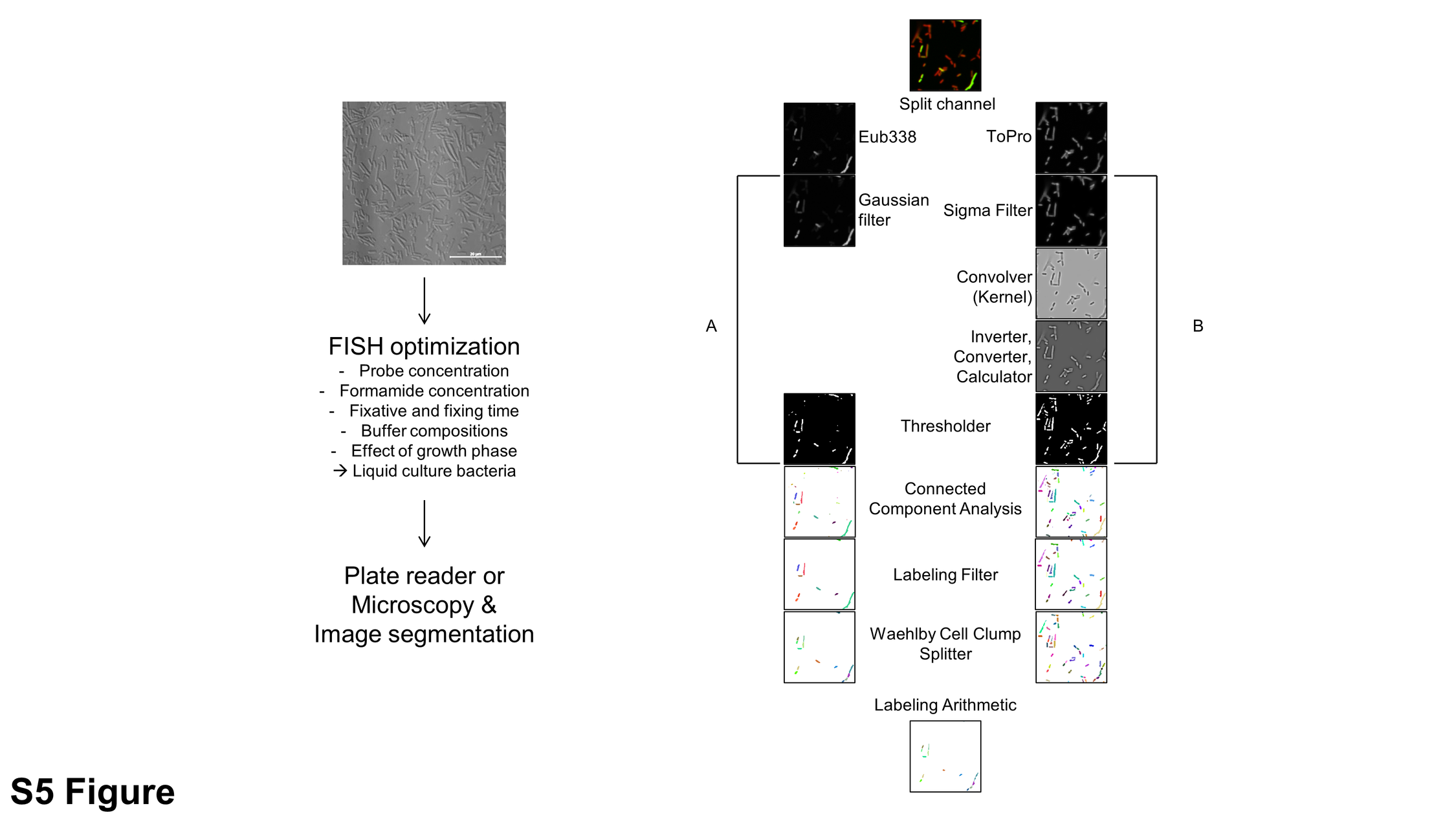

Supplement: S5 Fig — Flow-chart of the image segmentation pipeline for the quantitative analysis of the impact of various parameters on the FISH staining efficiency of singular bacterial type strain stainings. On the left an overview of the varied parameters during method optimization is given. On the right-hand side an overview of the image segmentation procedure performed with the KNIME image analysis platform is provided. The complete analysis pipeline and example images are provided at the KNIME hub (https://hub.knime.com/; see material and methods). (A) and (B) refer to separate analysis routines for the Eub338 and TO-PRO signals. (TIF) [file pone.0247376.s005.tif]

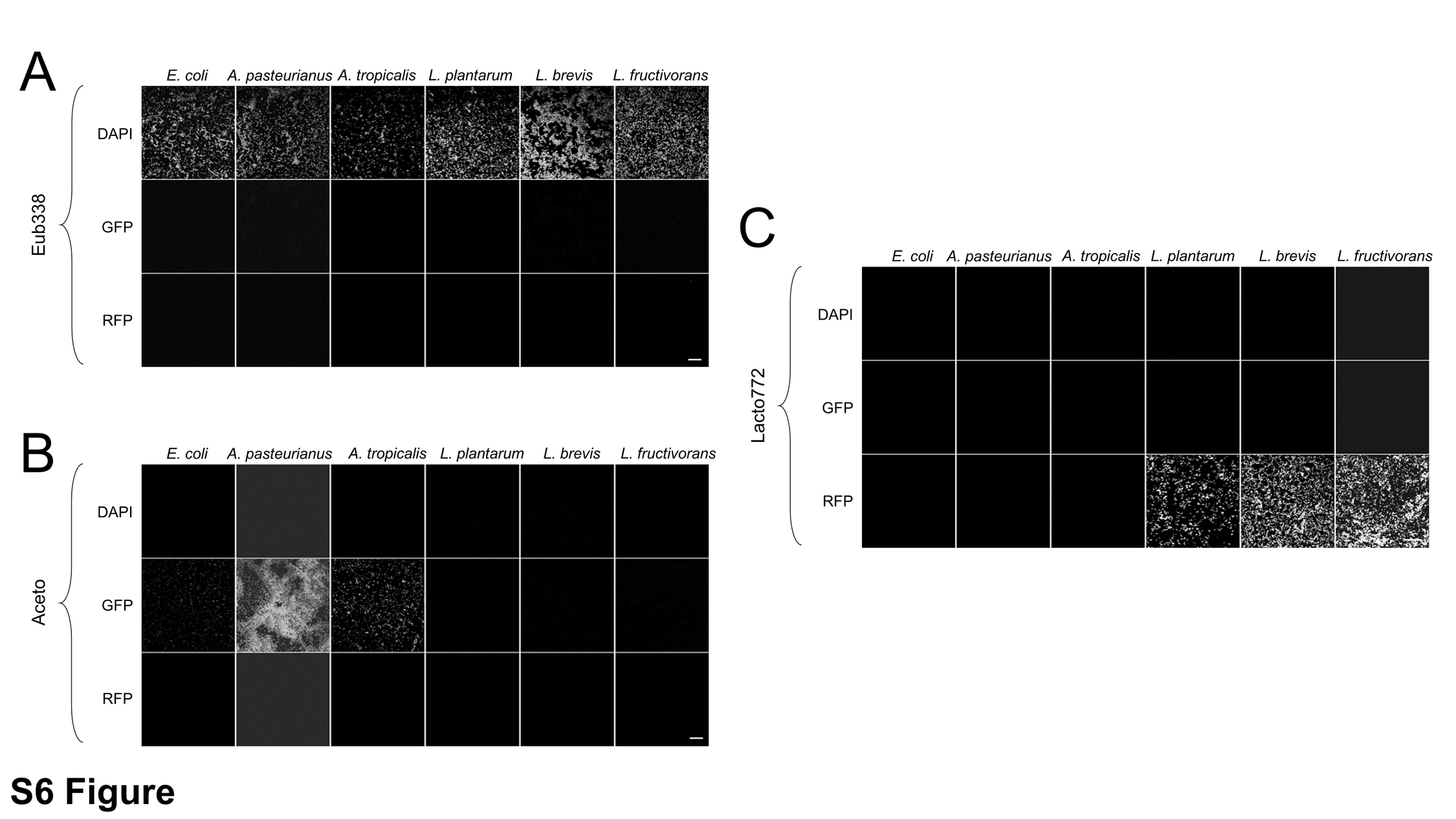

Supplement: S6 Fig — FISH was performed with PFA-fixed bacterial cell suspensions of E. coli, A. pasteurianus, A. tropicalis, L. plantarum, L. brevis, and L. fructivorans with the three different probes Eub338 (blue–detection in the DAPI channel), Aceto (green–detection in the GFP channel), and Lacto722 (red–detection in the RFP channel) (4 μM/probe) and 40% formamide for three hours at 46°C. Lactobacillus strains were treated with 10 mg/ml lysozyme for 15 minutes at 37°C prior to the hybridization. While each bacterial species sample was only stained with one probe (A: Eub388, B: Aceto, C: Lacto722) all detection channels were imaged with constant settings in order to test for potential fluorescence bleed-through. The figure shows representative examples from at least three separate experiments. Scalebar represents 20 μm. (TIF) [file pone.0247376.s006.tif]

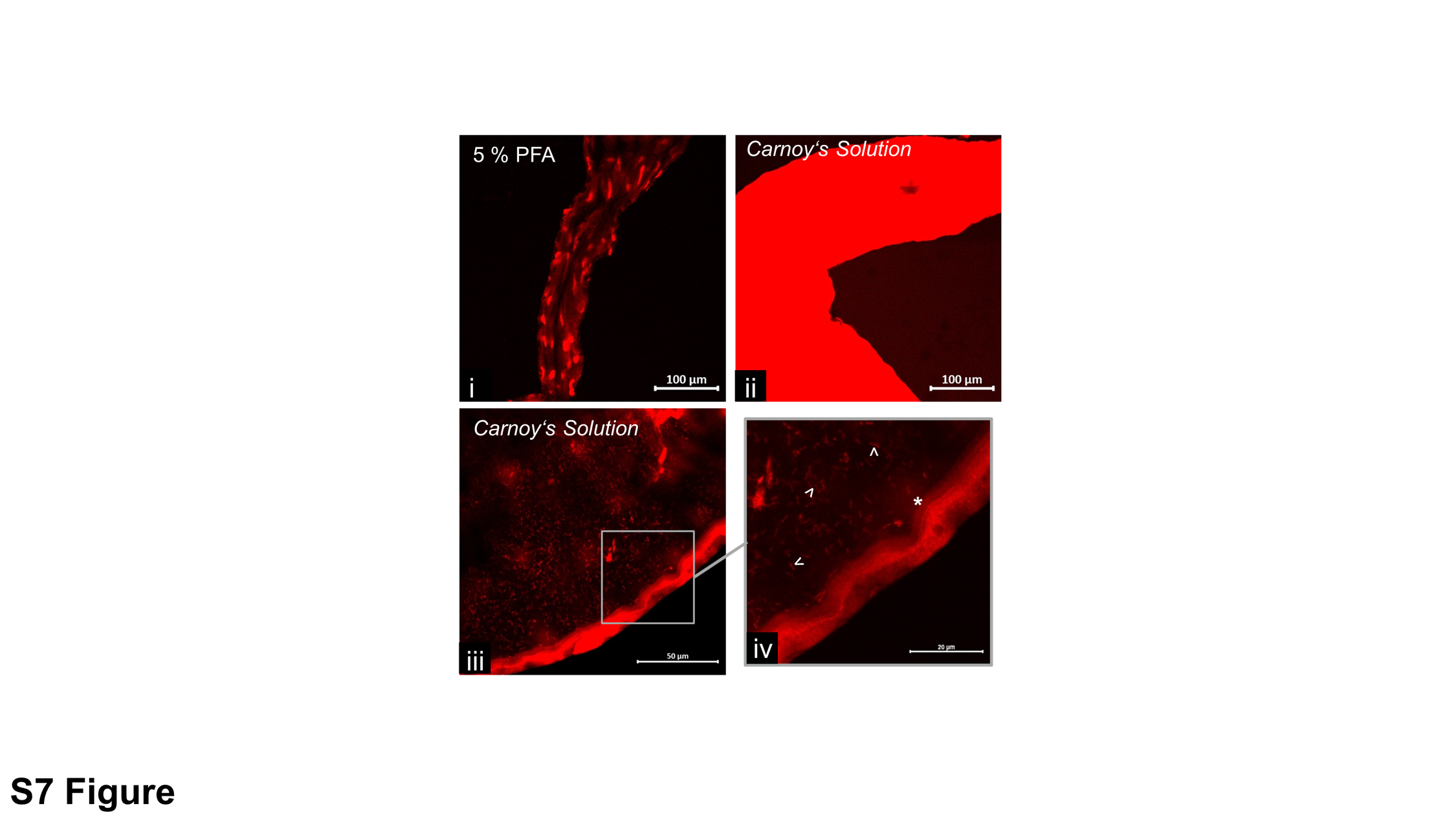

Supplement: S7 Fig — Larval Drosophila guts were fixed for 15 minutes using PFA (i) and for 5 minutes with Carnoy’s solution (ii-iv). DNA was stained with TO-PRO-3. (i) and (ii) were imaged using the same microscope settings and show the much higher staining intensity using the Carnoy’s solution. (iii) TO-PRO-3 stained bacteria in larval Drosophila gut lumen. (iv) Zoom-in of (iii) showing gut bacteria marked by arrowheads. The asterisk highlights the mucosa layer. Scalebars represent 100 μm (i-ii), 50 μm (iii), and 20 μm (iv). (TIF) [file pone.0247376.s007.tif]

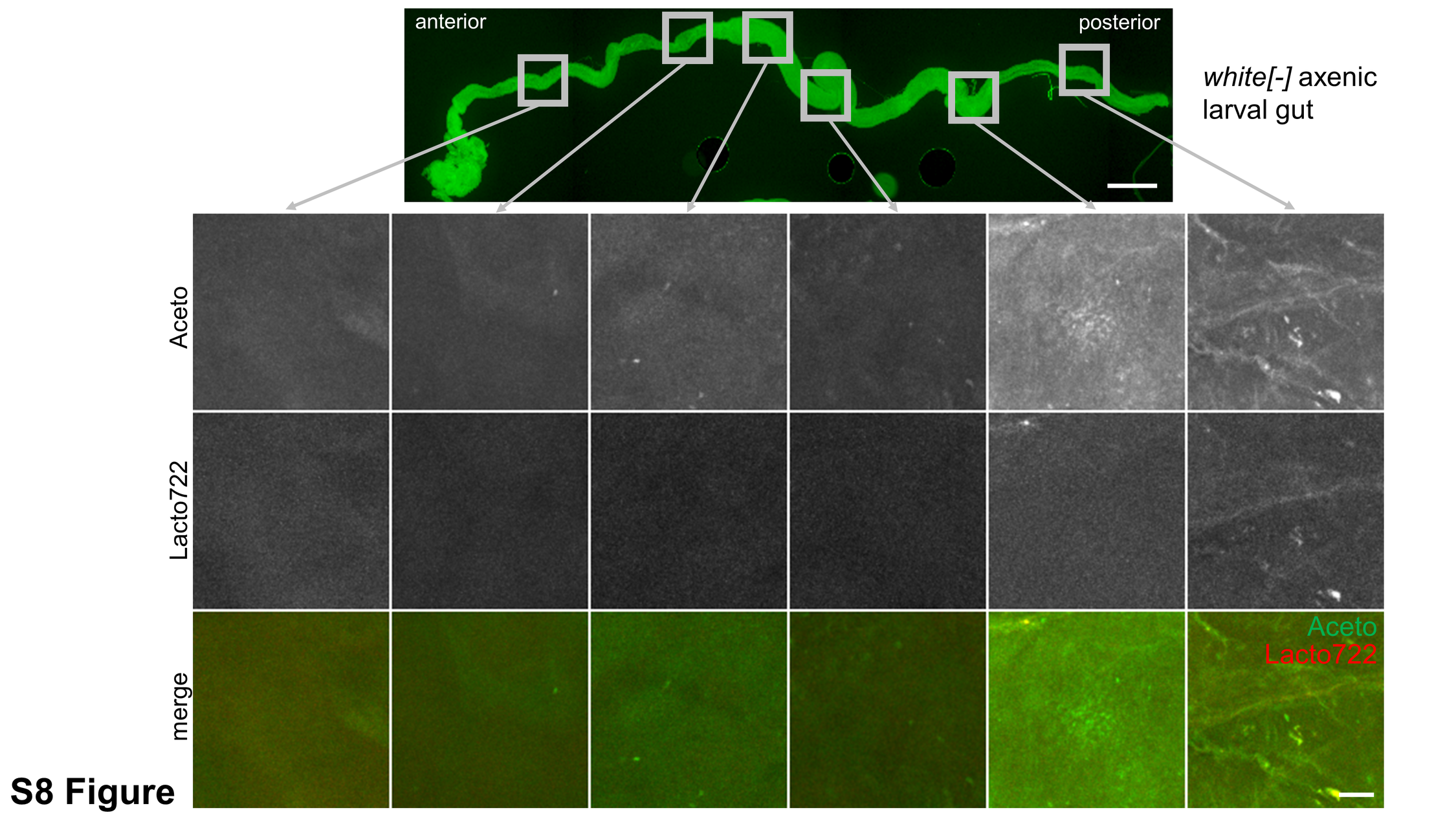

Supplement: S8 Fig — FISH was performed with axenic larval Drosophila guts using the genera-specific probes Aceto (green) and Lacto722 (red) (4 μM/probe) and 40% formamide for 16 hours at 46°C. Prior to hybridization, larval guts were treated with 10 mg/ml lysozyme for 15 minutes at 37°C. An overview of the entire gut was imaged and detailed zoom-ins of six different regions are shown. The figure shows representative examples from at least three separate experiments. In each experiment 5 to 10 guts per condition were dissected. Scalebars represent 500 μm (overview) and 10 μm (zoom-ins). (TIF) [file pone.0247376.s008.tif]

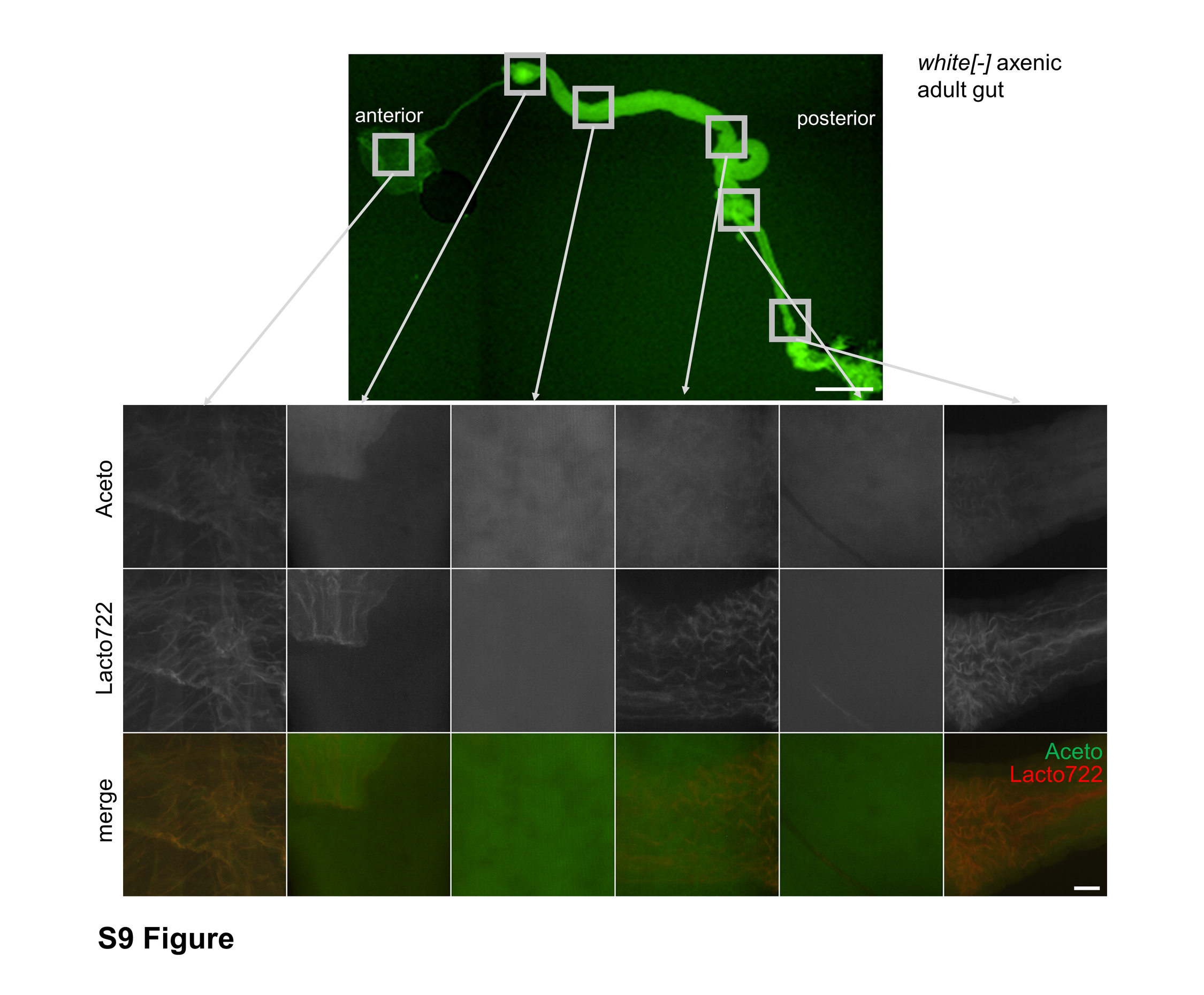

Supplement: S9 Fig — FISH was performed with axenic adult Drosophila guts using the genera-specific probes Aceto (green) and Lacto722 (red) (4 μM/probe) and 40% formamide for 16 hours at 46°C. Prior to hybridization, guts were treated with 10 mg/ml lysozyme for 15 minutes at 37°C. An overview of the entire gut was imaged and detailed zoom-ins of six different regions are shown. The figure shows representative examples from at least three separate experiments. In each experiment 5 to 10 guts per condition were dissected. Scalebars represent 500 μm (overview) and 10 μm (zoom-ins). (TIF) [file pone.0247376.s009.tif]
